# Home-Based Telehealth Exercise Intervention in Early-On Survivors of Childhood Acute Lymphoblastic Leukemia: Feasibility Study

**DOI:** 10.2196/25569

**Published:** 2021-06-16

**Authors:** Genevieve Lambert, Nathalie Alos, Pascal Bernier, Caroline Laverdière, Dahlia Kairy, Kenneth Drummond, Noémi Dahan-Oliel, Martin Lemay, Louis-Nicolas Veilleux

**Affiliations:** 1 Sainte-Justine University Health Center Montreal, QC Canada; 2 Department of Surgery-Division of Experimental Surgery Faculty of Medicine and Health Sciences McGill University Montreal, QC Canada; 3 Département de Pédiatrie Faculté de Médecine Université de Montréal Montreal, QC Canada; 4 École de Réadaptation Université de Montréal Montreal, QC Canada; 5 Centre for Interdisciplinary Research in Rehabilitation of Greater Montreal Montreal, QC Canada; 6 Research Institute of the McGill University Health Centre Montreal, QC Canada; 7 School of Physical & Occupational Therapy Faculty of Medicine and Health Sciences McGill University Montreal, QC Canada; 8 Shriners Hospital for Children - Canada Montreal, QC Canada; 9 Département des Sciences de l'Activité Physique Faculté des Sciences Université du Québec à Montréal Montreal, QC Canada; 10 Motion Analysis Center Shriners Hospital for Children - Canada Montreal, QC Canada

**Keywords:** exercise therapy, rehabilitation, acute lymphoblastic leukemia, intervention study, telehealth, mobile phone

## Abstract

**Background:**

Acute lymphoblastic leukemia is the most common type of pediatric cancer. Acute lymphoblastic leukemia causes an altered bone mineral homeostasis state, which can contribute to osteopenia, and bone fractures, most commonly vertebral fractures. With the increasing number of childhood cancer survivors, late adverse effects such as musculoskeletal comorbidities are often reported and are further influenced by inactive lifestyle habits. Physical activity has been shown to increase the mechanical workload of the bone, mitigating bone impairment in other cancer-specific populations.

**Objective:**

This interventional pilot study aims to investigate the use of telehealth to deliver a home-based exercise intervention for early-on survivors of bone marrow–related hematological malignancies and to assess its impact on survivors’ musculoskeletal and functional health.

**Methods:**

We aimed to recruit a group of 12 early-on survivors of acute lymphoblastic leukemia, within 6 months to 5 years of treatment, to participate in and complete the proposed telehealth intervention with a parent. The 16-week intervention included 40 potential home-based physical activity interventions supervised by a kinesiologist through a telehealth internet platform, with monthly progression. Patients were recruited to the cohort if they were able to participate in the intervention during the first month (minimum 12 weeks of intervention). Evaluation before and after the intervention protocol highlighted differences in functional capacities and musculoskeletal health of patients using mechanography, peripheral quantitative computed tomography, 6-minute walk test, and grip force test.

**Results:**

The recruitment rate for the intervention was low (12/57, 21% of contacted patients). Of 12 patients, 3 were excluded (1=relapse, 1=failure to meet technical requirements, and 1=abandoned). The 9 patients who completed the intervention (6 girls; mean age 10.93, SD 2.83 years; mean BMI 21.58, SD 6.55 kg/m^2^; mean time since treatment completion 36.67, SD 16.37 months) had a mean adherence of 89% and a completion rate of 75%. In addition, these patients showed functional improvements in lower limb muscle force and power as well as in the 6-minute walk test distance. Participants also showed improved bone health after the intervention on the following parameters: bone mineral content, stress-strain index, total and cortical cross-sectional area at the 14% site (*P*=.03, *P*=.01, *P*=.01, and *P*=.001, respectively) and 38% site of the tibia (*P*=.003, *P*=.04, *P*=.001, and *P*=.003, respectively).

**Conclusions:**

High adherence and participation rates suggest that telehealth is a feasible method to deliver exercise interventions to young early-on survivors of acute lymphoblastic leukemia. The proposed intervention seems promising in providing benefits to patients’ functional performance and bone health, but a large-scale study is needed to confirm this assumption.

## Introduction

### Background

Acute lymphoblastic leukemia is the most common type of cancer among the pediatric population. Over the past 50 years, the survival rates for pediatric hematological malignancies have increased significantly from nearly 0 to >80% because of the scientific advancements and improved therapeutic protocols [1]. Consequently, an increasing number of survivors are likely to experience long-term effects of the disease, treatment toxicities, and increased inactive lifestyles [2,3]. Furthermore, the specific immune cells at the origin of the acute lymphoblastic leukemia originate from stem cells in the bone marrow. Therefore, it is not surprising that the disease, treatments, and modified lifestyle habits contribute to comorbidities and late adverse effects of the musculoskeletal system in long-term survivors [4]. Among these comorbidities, a decrease in muscle strength [5], bone mass [6,7], and an increased prevalence of vertebral fractures [8,9] have been reported. These musculoskeletal adverse effects can be apparent on initial diagnosis, increase in severity during the acute phase of treatment [9], and remain present [10] or appear during remission [11] and survival [12,13].

Physical activity and exercise provide physiological and mechanical stimulation that are beneficial for muscle and bone health [14] and the cardiovascular system [15]. Specific types of exercises, such as plyometric (defined as high impact, eg, jumping) and resistance exercises, have been shown to decrease bone impairments in other cancer populations with bone-specific deficits (breast cancer and prostate cancer) [16]. Therefore, an exercise rehabilitation intervention administered to early-on survivors of hematological malignancies, with plyometric and resistance exercises aiming at improving muscle function and bone strength, could limit the musculoskeletal late adverse effects reported in long-term survivors.

Medical follow-up visits for survivors of acute lymphoblastic leukemia are generally performed 1-4 times per year in pediatric oncology centers, limiting the feasibility of an in-clinic exercise intervention. In this regard, studies have shown that patients and survivors would rather exercise at home, school, or a fitness club, than at a hospital or physiotherapy clinic [17,18]. For these reasons, home-based exercise interventions are considered the most appropriate intervention method for this population. Only a few studies have addressed the effects of home-based exercise interventions on muscle function of children with acute lymphoblastic leukemia in maintenance or early-on survivorship, with equivocal results. In a study by Tanir and Kuguoglu [19], a home-based physical exercise intervention was provided to the patients for 3 months, with muscle strength, aerobic, and stretching exercises. The results showed significant improvements in flexibility and muscle and cardiopulmonary functions. Similarly, a study by Esbenshade et al [20] yielded similar results for a 6-month home-based exercise intervention. In contrast, studies by Marchese et al [21] and Hartman et al [22] showed only minor improvements in muscle function (increased knee extensors and ankle dorsiflexor strength) and no improvement in cardiopulmonary function [21] and bone health [22]. Both studies that showed significant improvements in physical fitness, that is, the studies by Tanir and Kuguoglu [19] and Esbenshade et al [20], reported a high adherence rate (mean 82%, SD 7%), whereas the studies by Marchese et al [21] and Hartman et al [22] reported low adherence rates. Taken together, the results of these studies suggest that patients with acute lymphoblastic leukemia in the maintenance or early-on survivorship can benefit from a home-based exercise intervention but that high adherence rates are required to achieve significant improvements in the musculoskeletal system.

Adherence rates tend to be lower in the absence of supervision in home-based exercise interventions. For example, both studies (Marchese et al [21] and Hartman et al [22]) that showed minimal or no effect of the home-based exercise intervention reported a minimal follow-up approach (between biweekly and monthly phone calls with the sole objective to assess adherence), likely resulting in the reported low adherence rates. In contrast, both studies that showed improvements following the home-based exercise intervention had set up a stringent supervision (weekly or biweekly follow-up calls to discuss factors of adherence), resulting in high adherence rates [19,20]. In this regard, a recent literature review suggests that home-based exercise interventions with telehealth supervision improve adherence rates compared with no supervision [23] because of patients receiving positive reinforcement [24], improving on the exercise technique [24] and feeling self-efficient [25]. Another potential positive impact of supervision is the greater overall volume of exercise achieved during individual sessions, which can be associated with better structured and controlled exercised sessions under supervision compared with no supervision [26]. These observations suggest that supervision by health care providers during home-based exercise training may help patients achieve higher adherence rates and obtain additional benefits compared with no or minimal supervision.

Telehealth is defined as a method of delivering health interventions (eg, physical activity, nutritional, and psychological counseling) or follow-ups from a remote location through information technologies (eg, the internet). The research field associated with telehealth has experienced significant growth over the past 10 years, leading to an exponential increase in its application in light of the current COVID-19 global pandemic. Over the past decade, telehealth has been shown to be efficient in achieving high adherence rates compared with traditional home-based exercise intervention in patients with musculoskeletal, neurological, cardiorespiratory, and various other conditions [27,28]. However, to our knowledge, this study is the first to report the feasibility of implementing a home-based exercise intervention with telehealth supervision in early-on survivors of pediatric cancer.

### Objectives

The primary aim of this study is to assess the feasibility of implementing a home-based exercise intervention with telehealth-based supervision for early-on survivors of acute lymphoblastic leukemia. Telehealth can be administered using various technologies. Desktops, laptops, tablets, and smartphones have the ability to provide and receive telehealth services. Although tablets and laptops provide mobility options compared with a desktop solution, this study was designed to favor accessibility; therefore, families could select the technology of their choice to receive the intervention, be it a fixed desktop or a mobile phone and tablet. In addition, as having companions for exercising has been identified as a facilitating element in adherence [17], we grouped patient-parent pairs with one or two other pairs. The feasibility of the pilot intervention was evaluated by assessing the completion and adherence rate of patients, in addition to the occurrence of training adaptation because of participants’ pain and adverse events. It is hypothesized that direct supervision, possible through telehealth technologies, will lead to an adherence rate of 80% and a completion rate of 75% [15]. The secondary aim of this study is to explore the effects of the intervention on functional performance, muscle function, and bone health. It is hypothesized that the intervention will lead to improvements in musculoskeletal and cardiopulmonary function.

## Methods

### Study Design and Recruitment

This prospective pre- and postintervention cohort pilot study was initiated in 2018 at Sainte-Justine University Health Center to assess the feasibility of home-based exercise interventions in early-on survivors of hematological bone marrow–related malignancies who have been treated under Dana-Farber Cancer Institute–acute lymphoblastic leukemia 2005 or 2011 protocols. As the research design was a pilot interventional study, no sample size calculation was made, and a convenience sample of 10 participants for intervention completion was set as the aim. The initial inclusion criteria were diagnosis of acute lymphoblastic leukemia or B lymphoblastic lymphoma, age between 6 and 18 years, and within 6 months to 5 years of treatment completion. Exclusion criteria were unresolved fractures, unresolved avascular osteonecrosis, and bone marrow transplantation as part of their treatment; physical or functional impairment at the time of recruitment was excluded. If patients had no or unstable internet connection, they would further be excluded. Owing to recruitment challenges for the first cohort, a first amendment was submitted to the ethical review board to increase the oldest age of eligibility from 10 to 14 years. Owing to recruitment challenges for the second cohort, a subsequent amendment was submitted to further increase the age range from 6 to 18 years, in addition to modifying the criterion of time since treatment completion from 6 months to 2 years to 6 months to 5 years. Patients could be included in the cohort if they participated in the exercise intervention within the first month of the intervention to receive between 12 and 16 weeks of the intervention.

Patients were screened for eligibility by the hematology oncology service medical team (nurses and physicians) at Sainte-Justine University Health Center.

Healthy age- and sex-matched participants were retrospectively included as controls for muscle function and bone analyses. Owing to the retrospective nature of this cohort, participants in the control group were not subjected to the intervention, and muscle and bone data were available at only one time point. These controls were drawn from our local historical database, including healthy siblings of patients and children of hospital staff who were part of a previous study. Control participants were selected based only on sex and age to avoid any selection bias, for example, in selecting patients that would decrease the difference between controls and patients in muscle and bone parameters.

The Sainte-Justine University Health Center institutional review board approved this study (2018-1555: e-S@@VIE). Parents of patients aged <18 years provided signed informed consent, and patients aged between 6 and 17 years provided informed consent. Families were contacted via phone to provide details of the project and check for interest. If they were interested in the study, a baseline evaluation was performed.

### Study Procedures

The study procedure was divided into the following four phases: (1) baseline evaluation, (2) home-based visit, (3) intervention, and (4) postintervention evaluation.

#### Baseline Evaluation

After informed consent or assent was provided, patients completed baseline (and postintervention) measurements at 2 pediatric health care centers in the Montreal area: Sainte-Justine University Health Center and Shriners Hospital for Children, Canada. The baseline and postintervention visit schedule followed the same pattern: at Sainte-Justine University Health Center, weight and height of the patients were measured, and 6-minute walk test (6MWT), upper limb grip force test, and lower limb mechanography were evaluated. All participants were assessed by the same trained evaluator (GL). At Shriners Hospital for Children, patients underwent bone imaging testing (peripheral quantitative computed tomography [pQCT]).

#### Home-Based Visit

Following the baseline evaluation, a kinesiologist visited the families at their homes to help them prepare for the intervention. The kinesiologist delivered the following materials to the patients: an exercise step, a training elastic, a weighted 5-pound ball, and a training watch (Polar A370, Polar Electro Oy 2020, Polar FlowSync 3.0.0.1337) and its charger. At the same time, an assessment was performed for the suitability and safety of the space (1.8 m^2^ of free space required). Support was provided for the installation of the software (for the watch and the videoconferencing system) on their own technologies (tablet, laptop, and computer) [29].

#### Intervention

All home-based exercise interventions were performed using a teleconferencing system (Zoom license Pro, Zoom Video Communications, Inc) with a kinesiologist at the hospital center and the study patients and their parents in their homes. This system was chosen because it provides encrypted communication between the kinesiologist and the families, which is compliant with the Canadian federal law about the privacy of companies, the *Personal Information Protection and Electronic Documents Act*. Families were sent an email 24 hours before every training with the link to connect to the virtual *meeting room* for their respective group. Interventions were live interactions that enabled direct supervision and immediate correction or adaptation of the exercise intervention when needed (for safety purposes). Study patients were divided into three groups of two families and two groups of three families based on language (English or French), age, and availability. Three cohorts were supervised at different time points (May-August 2018, January-April 2019, and September-December 2019). The original 16-week intervention included a progression every 4 weeks. Weeks 1-4 involved two sessions of 35 minutes per week. There was an additional 5 minutes of training per session during weeks 5-8, that is, two sessions of 40 minutes per week. During weeks 9-12, one session was added every week, that is, three sessions of 40 minutes per week. Finally, during weeks 13-16, an additional 5 minutes was added to each of the three sessions per week, bringing the duration to 45 minutes per session [30]. For the first 8 weeks, the training sessions were held on weekday evenings, and for the last 8 weeks, a third training session was added either on weekday evenings or on a weekend day. The general organization of a training session was as follows: a 5-minute warm-up, followed by whole-body resistance exercises (of progressive duration through the 16-week intervention), and finally, 5-minute stretching. The resistance exercises part of the training consisted of whole-body exercises (eg, push-ups, squats, and deadlifts) combined with plyometric exercises (eg, drop jumps, hopping, and jumping lunges). The training sessions and exercises were adapted according to the participants’ pain reports. Pain was evaluated at the beginning and end of the session, as well as during sessions when pain was present at the beginning of the session. Pain was rated on a scale from 0 to 10 (Numerical Rating Scale-11 [31]), a description of the perception of pain (sensation and location) and its evolution through time and movement. The adaptations were personalized according to the location and intensity of the pain. For example, patients with moderate knee pain would not do impact exercises such as *high-knees jogging* but would do low impact exercises such as *walking* or no impact exercises with *chair squats* or *calves raise* instead.

#### Postintervention Evaluation

The same evaluations assessed at baseline were performed at the end of the home-based exercise intervention, in the same context as the baseline evaluation.

### Outcome Measures

#### Primary Endpoints: Feasibility

To determine the feasibility of administering a home-based intervention through telehealth to this population, recruitment rate, reasons for declining participation, the mean adherence rate and the completion rate to the intervention were computed. The recruitment rate was defined as the number of consented patients divided by the contacted potential patients. The adherence rate was defined as the number of sessions attended by the patients divided by the total number of possible sessions. Individual reasons for missing sessions have been reported. In addition, the specific information technologies (tablet, mobile phone, or computers) used for the interventions were reported for each household. Completion rate was defined as the number of patients who completed the intervention divided by the total number of patients who consented. The total number of training sessions with modified exercises owing to participant’s pain was recorded. Finally, the nature and extent of adverse events during the training sessions were assessed by the kinesiologist according to the type and severity of events defined as potentially sequelae in a study by Ory et al [32].

#### Secondary Endpoints: Functional Performance and Bone Health

##### Muscle Parameters: Mechanography and Grip Force Test

Mechanography is a technique developed to investigate lower limb muscle function using a ground reaction force–measuring platform (Leonardo Mechanograph Ground Reaction Force Plate; Novotec Medical GmbH). Forces were recorded over time at a sampling rate of 800 Hz. All parameters reported here were derived from these force-time data using proprietary software (Leonardo Mechanography GRFP Research Edition software, version 4.2-b05.53-RES; Novotec Medical GmbH).

In total, two tests were performed using mechanography: the single two-legged jump (S2LJ) test for maximal power and the multiple two-legged hopping (M2LH) test for maximal force. The methodology is described in detail elsewhere [33,34]. Briefly, the S2LJ is a countermovement jump, and maximal power (kW) and maximal relative power (W/kg) are the main outcome parameters for this test. The M2LH test consists of hopping on the forefeet with stiff knees and without the heels touching the ground (similar to rope skipping). The M2LH provides information on the near-maximal ground reaction forces during eccentric contraction generated by patients. Relative muscle force (calculated in multiples of body weight) has been identified as the main parameter of this objective, as it is strongly associated with bone strength [35]. The participants were asked to perform three trials for each test. A trial for the S2LJ consists of performing one jump, whereas a trial for M2LH consists of 10 consecutive hops. If the trials were not performed properly, an additional two trials were attributed to acquiring three valid test results. The trials with the highest peak power and peak force for S2LJ and M2LH, respectively, were selected for analysis.

The grip force test was performed using a handgrip dynamometer (Jamar Hand Dynamometer, Jamar Technology Inc.), which evaluates the maximal isometric force of the upper limb muscles. The patients were instructed to stand, feet shoulder-width apart, with arms in a neutral resting position on both sides of the body. They were then given a dynamometer that had previously been adjusted to an individual patient’s hand. Finally, patients were instructed to press the handle as hard as possible until they were told otherwise. The test was performed on one arm at a time; both sides were repeated twice, and the best result of both sides was selected as the participant’s result. The dynamometer provides force data in kilograms, and the evaluator was instructed to round the result to the nearest kilogram [36]. Scores were calculated based on grip force test reference data to compare the patients’ results with a healthy sex- and age-specific population [37].

##### Cardiopulmonary Function: 6MWT

The 6MWT evaluates the ability of an individual to maintain a moderate level of physical activity over a 6-minute period [38]. Therefore, the result of the 6MWT is a reflection of the patient’s daily activities [39]. The 6MWT correlates significantly with maximal oxygen uptake in typically developing children as well as in patients and survivors. This indicates that these two tests measure related functional capacities [39-41]. Study patients followed the instructions from the *American Journal Respiratory and Critical Care Medicine* published guidelines (2002): to walk back and forth in a hallway between two cones distanced by 30 m for 6 minutes as fast as possible at a pace that would make them tired by the end of the walk; encouragement and feedback are given every minute. During the test, patients were allowed to rest if needed. Expected results equations are available for calculating the percentage of age- and sex-specific norms [42]. The 6MWT has been shown to be reliable and valid in typically developing children (2-4 weeks apart between test and retest) and obese children (same-day test-retest), with a reliability reported from 0.73 to 0.949 [40,43]. Expected results were used to compare the results of the patients with a healthy sex- and age-specific population (*equations to predict the 6-minute walk distance in children and adolescents* [42]).

##### Bone Health: pQCT

pQCT was performed on the left tibia, unless there was a medical history of fracture of the bone, using the Stratec XCT2000 (Stratec Inc). This method is described in detail elsewhere [44,45]. The lower leg was scanned at 4% (metaphysis and trabecular bone), 14% (metaphyseal-diaphyseal transition site and cortical bone), 38% (diaphyseal transition site and cortical bone), and 66% (muscle parameters scan and midsection of the gastrocnemius muscles, therefore being the largest outer calf diameter [46]) of tibia length, measured as the distance from the reference line. The tomography images were then ranked using the movement artifact scale from 1 to 5, 1 being an image without the artifact and 5 being there was too much movement to have a proper image. Scans scoring ≤3 were deemed usable. If the scan scored 4 or 5, the test was redone [47].

The main bone outcome parameters of pQCT analysis were measured at the 4%, 14%, and 38% sites of the tibia length, with 4% being the distal part of the tibia. The following parameters were measured: total bone cross-sectional area (CSA; mm^2^), cortical bone CSA excluding marrow space (mm^2^), bone mineral content (BMC) per millimeter of cross-sectional slice thickness (mg/mm), total volumetric bone mineral density (vBMD; mg/cm^3^), trabecular CSA (mm^2^; 4% site only), trabecular vBMD (mg/cm^3^; 4% site only), cortical vBMD (mg/cm^3^), and polar stress-strain index (SSI; assessed as a surrogate of bone strength; mm^3^). The two main pQCT muscle outcome parameters were measured at the 66% site: muscle CSA (unit: mm^2^; 66% site) and muscle density (unit: mg/cm^3^; 66% site) [32].

### Statistical Analysis

As this study was a pilot study to investigate the feasibility, no sample size calculation was performed. The normality of the data was tested using the Shapiro-Wilk test (n=9) [48]. The means and SDs were reported when the data were normally distributed, and the median and range were reported when the normality assumption was violated.

To assess feasibility, recruitment, completion, and adherence rates were analyzed. A one-sample Wilcoxon signed-rank test was performed on the adherence rate of patients with a set threshold of 80%, based on the hypothesis. The threshold was based on a study involving home-based distance-delivery exercise interventions administered to patients with acute lymphoblastic leukemia in remission, which showed an 80% adherence rate for a 75% completion rate [15].

To determine the effect of the exercise intervention on patients’ functional and musculoskeletal health, pre- and postintervention test results from the pQCT, mechanography, grip force, and 6MWT were compared using the paired-samples two-tailed *t* test when the data were normally distributed and the related-sample Wilcoxon signed-rank test when they were not. In addition to these pre- to postanalyses, postintervention results of pQCT, mechanography, and grip force [37] were compared with sex- and age-matched typically developing controls using independent-samples *t* tests (for normally distributed parameters) and the independent-samples Mann-Whitney *U* test (for parameters not normally distributed). Additional analysis included a one-sample *t* test analysis to determine if the mechanography results were clinically significant by comparing patients’ change in lower limb muscle function with the minimal detectable difference reported by Veilleux et al [33]. For the 6MWT, an independent-samples *t* test was performed on the distance traveled after the intervention and expected distance of the 6MWT from sex- and age-related calculations [42]. Patients’ changes in 6MWT distance were compared in a one-sample *t* test analysis with the SE of 15 m reported by Li et al [40] to establish if the results were deemed clinically significant.

To assess the muscle-bone functional unit, a Spearman correlation was performed for nonnormally distributed parameters. The correlation between maximal force (absolute; N) and BMC at 14% of the tibia was established for both pre- and postintervention patient-related data as well as for the typically developing controls [35].

All statistical tests were performed using Predictive Analytics Software Statistics software version 24.0 (SPSS Inc), with the CI and significance level preset at 95% and .05, respectively.

## Results

### Feasibility and Baseline Characteristics

**Figure 1:**
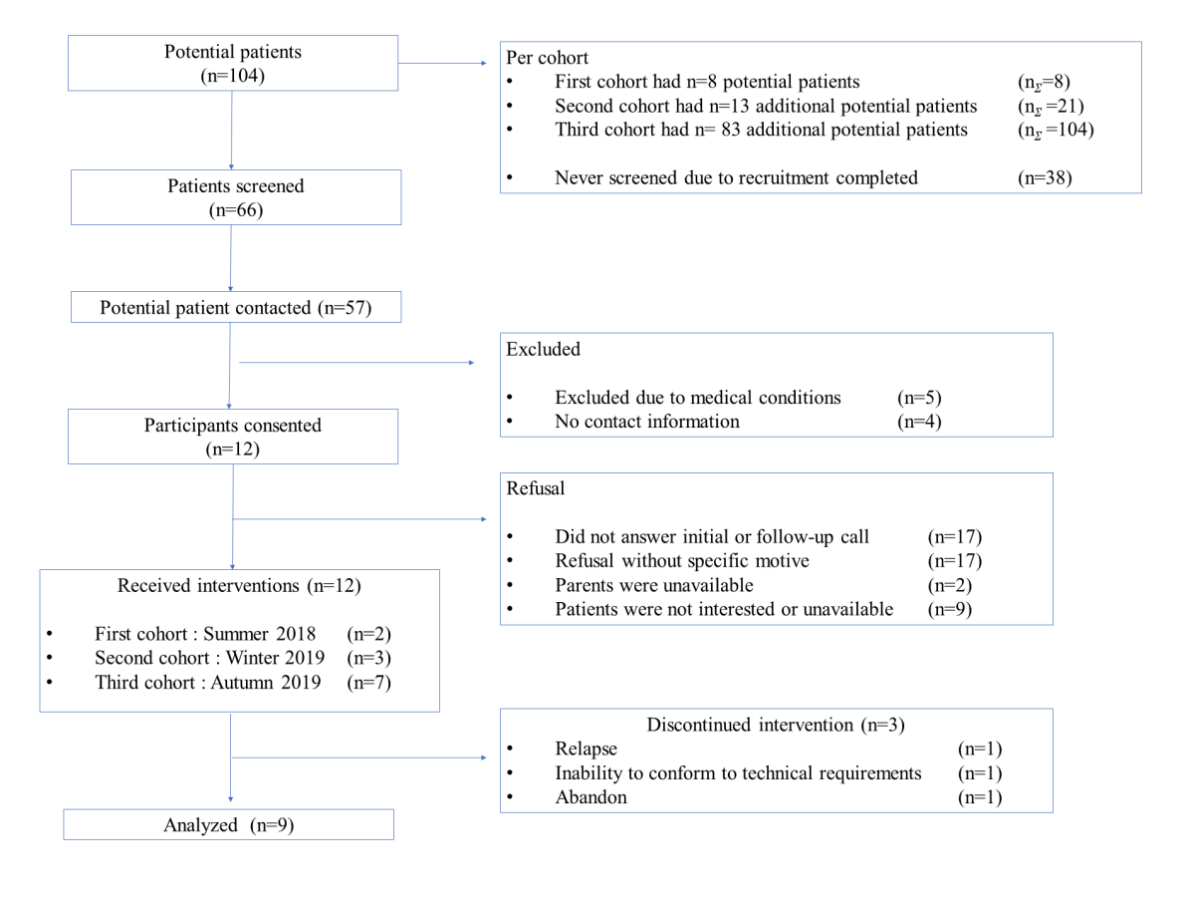
Recruitment process flowchart; n represents the number of individuals in the sampling; n_Σ_ represents the summation of all potential participants at that timepoint.

The recruitment flowchart is shown in Figure 1. A total of 104 patients aged 6-17.1 years within 5 years of complete remission were considered as potential participants. Of the 57 potential participants who were contacted, 12 patients (21%; 9 girls) provided informed consent or assent (Table 1 provides participant clinical information). The specific motive to decline participation was recorded in 27% (12/45) of refusals: parents’ overloaded schedules (n=2); the patients were deemed too active by their parents, as they engaged in other physical activities multiple times per week (n=4); the patients did not want to come to the hospital for the evaluation (n=2); or the patients did not find the idea of an organized training session interesting (n=3).

**Table 1:** Clinical information.

Anthropometric and clinical parameters		Baseline (n=9)	Postintervention (n=9)	Controls (n=9)
Age (years), median (range)		9.17 (8-14.5)	9.5 (8.25-15.1)	9.87 (7.48-14.72)
Sex (female), n (%)		6 (75)	6 (75)	6 (75)
Height (cm), mean (SD)		143.27 (23.63)	145.19 (23.63)	146.03 (17.54)
Weight (kg), mean (SD)		46.92 (24.67)	47.88 (24.92)	40.71 (11.64)
BMI (kg/m^2^), mean (SD)		21.58 (6.55)	21.46 (6.53)	18.83 (3.39)
**Diagnosis, n (%)**				
	Acute lymphoblastic leukemia	8 (89)	—^a^	N/A^b^
	Lymphoblastic lymphoma	1 (11)	—	N/A
Prognosis, (SR:HR:VHR)^c^		6:2:1	—	N/A
Time since end of treatment (months), mean (SD)		36.67 (16.37)	—	N/A
Recurrence, n (%)		1 (11)	—	N/A
**Treatment protocol, n (%)**				
	DFCI-ALL^d^ 2005	2 (22)	—	N/A
	DFCI-ALL 2011	7 (77)	—	N/A
**Cumulative dose of glucocorticoids**				
	Dexamethasone, median (range)	352 (256-870)	—	N/A
	Prednisone, median (range)	390 (252-2199)	—	N/A
Cranial radiotherapy, n (%)		1 (11)	—	N/A
Duration of hospitalization during treatments (days), mean (SD)		45 (13)	—	N/A
**Musculoskeletal comorbidities during treatments, n (%)**				
	Vertebral fracture	4 (44)	—	N/A
	Osteonecrosis	1 (11)	—	N/A
	Nonvertebral fracture	2 (22)	—	N/A
	Osteoporosis	4 (44)	—	N/A
	Low bone mineral density	8 (89)	—	N/A
Received bisphosphonates, n (%)		4 (44)	—	N/A
Cumulative dose of zoledronic acid, median (range)		3.13 (1.70-4.05)	—	N/A
**Other comorbidities during treatments, n (%)**				
	Thrombosis	4 (44)	—	N/A
	Neuropathy	1 (11)	—	N/A
Home distance from health care center (round trip; km), median (range)		66 (7-72)	—	N/A

^a^Not reported.

^b^N/A: not applicable.

^c^SR:HR:VHR: standard risk:high risk:very high risk.

^d^DFCI-ALL: Dana-Faber Cancer Institute–acute lymphoblastic leukemia treatment regimen.

Of the 12 enrolled patients, 9 patients completed the 12- to 16-week intervention and had complete pre- and postintervention data sets, representing a 75% completion rate (Figure 1). Of the 12 patients, 3 did not complete the final evaluation because of technical issues (poor internet connection, n=1), relapse (n=1), or dropped out because of lack of interest (n=1). Of the 9 patients who completed the intervention, 5 had 40 potential training sessions and the others had 31, 32, 34, and 38 potential training sessions. Overall, the group’s median for adherence rate was 95% (range 70-98; *P*=.04), that is, an average of 33 sessions attended on 37 possible sessions. All participants required adaptations due to pain, on average, for 16 sessions (range 14-27), representing 48% of the training done. Table 2 illustrates the reasons for missing a training session and the overall proportion it represents.

**Table 2:** Reasons for participants’ absences to the exercise sessions (n=35)^a^.

Reason of absence	Values, n (%)
Patient acute musculoskeletal pain	6 (17)
Patients’ comorbidities or infections (eg, asthma or pneumonia)	8 (23)
Patients’ sickness (eg, cold, fever, flu, and so on)	10 (29)
Parents’ unavailabilities	3 (8)
Patients’ other activities (eg, school-related activities, sports, and so on)	6 (17)
Technical failure	2 (6)

^a^The total of missed sessions is 35 out of 335; the values presented as the absolute number of absence and their relative weights according to their respective reasons for absence.

With regard to the information technology used to receive the telehealth intervention, one family used a desktop computer connected to their television, resulting in a fixed setup; two families used a tablet; and six families used a laptop for interventions. Of the six households that used a laptop, three connected the device to the television to allow for a larger screen view. Mobile technology was also used outside home settings (ie, at the hotel during family vacation: n=2; at the house of family members such as divorced parents, grandparents, uncles, or aunts: n=3; or to benefit from outdoor settings: n=1). The kinesiologist provided most of the sessions within the hospital setting using a fixed system, except for six training sessions delivered outside hospital settings using mobile technology (laptop) for 2 weeks while on conference travel abroad.

The kinesiologist reported four occurrences of mild adverse events over 300 training sessions. The events were intervention related and resulted from falls (n=2) or missteps (n=2). All patients were able to resume training within minutes after the event had occurred. None of the patients had lasting effects, and it did not prevent patients from participating in any of the following sessions.

### Functional Performance and Bone Health

#### Muscle Parameters

**Figure 2:**
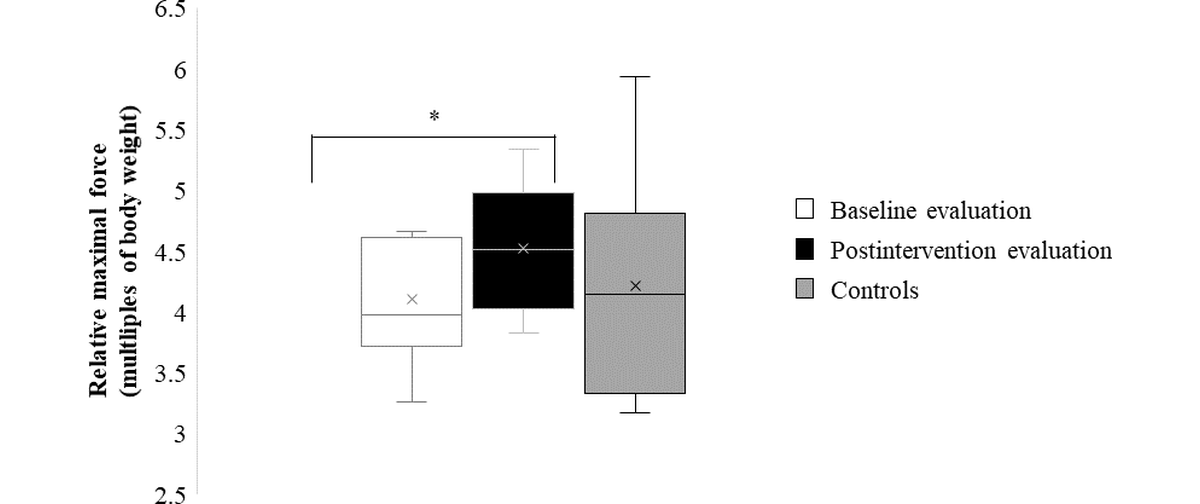
Box and whisker plots of the mechanography results; relative maximal muscle force at baseline, postintervention, and for controls. The “*” indicates paired-sample *t* test comparing baseline and postintervention data, significant at *P*=.05.

**Figure 3:**
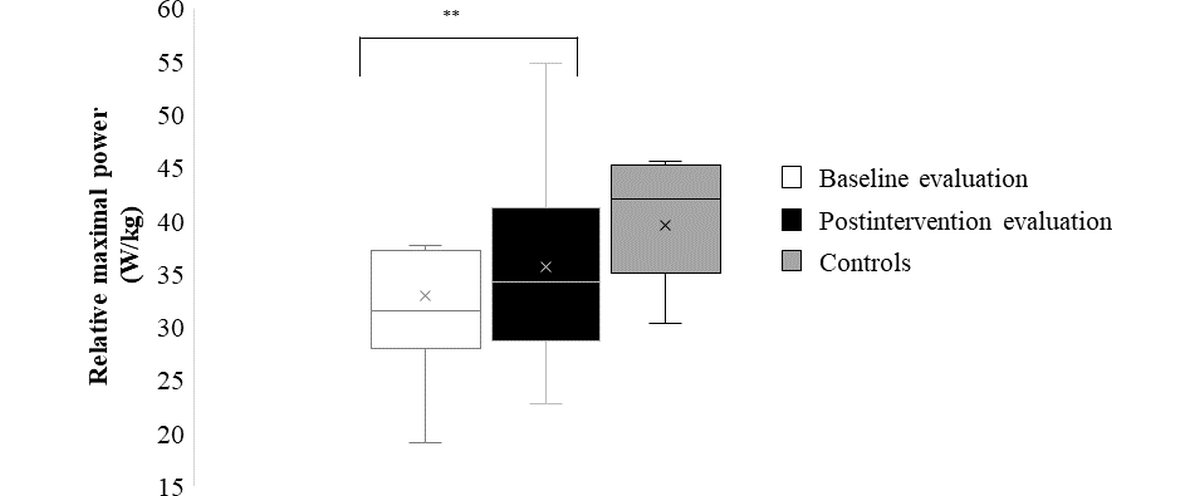
Box and whisker plots of the mechanography results; relative maximal muscle power at baseline, postintervention evaluation, and for sex- and age-matched controls. The “**” indicates paired-sample *t* test comparing baseline and postintervention data, significant at *P*=.002.

All functional performance parameters are reported in Table 3, except for relative maximal force and power of the lower limb, as illustrated in Figures 2 and 3. Lower limb muscle function showed a significant increase from pre- to postintervention for relative maximal force (11%; Figure 2), in addition to absolute (11%) and relative maximal power (9%; Figure 3). The absolute force data of the lower limbs showed no significant difference between pre- and postintervention. The analyses comparing postintervention mechanographic data of study patients with typically developing controls showed no significant difference for both relative force (*P*=.76) and relative power (*P*=.08).

**Table 3:** Functional outcomes.

Outcome			Baseline evaluation (n=9)		Postintervention evaluation (n=9)		Controls or expected results (n=9)		*P* value^a^		*P* value^b^
**Mechanographic parameters**											
	Absolute force (kN)^c^	1.17 (0.96-4.06)		1.60 (1.08-3.72)		1.63 (1.02-2.63)		.10		.73	
	Absolute power (kW)^c^	0.97 (0.66-3.03)		1.07 (0.72-3.14)		1.57 (0.95-2.73)		*.008* ^d^		.73	
**Hand dynamometer**											
	Grip test right (kg)	16.6 (8.4)		17.3 (7.7)		—^e^		.50		.16^f^	
	Grip test left (kg)	14.6 (8.7)		15.6 (7.7)		—		.52		.21^f^	
6-minute walking test distance (m)			593 (100)		646 (97)		598 (43)		*.01*		.90

^a^*P* values of paired-sample *t* test and Wilcoxon matched-pairs signed-ranked test comparing baseline and postintervention evaluations.

^b^*P* values of independent-samples *t* test and independent-samples Mann-Whitney *U* test comparing postintervention with control data.

^c^Parameters not normally distributed.

^d^Italicized *P* values denote significance (*P*<.05).

^e^Not available.

^f^*P* value of the paired-sample *t* test of the grip strength Z-scores comparing baseline and postintervention evaluations.

Absolute upper limb isometric grip force showed no significant difference between the pre- and postintervention results. Isometric grip force results showed that patients had lower grip force than normal (average *z* score right hand: preintervention −1.06, SD 0.66, *P*=.001; postintervention −0.73, SD 0.94, *P*=.05; and average *z* score left hand: preintervention −1.63, SD 0.86, *P*<.001; postintervention −1.19, SD 0.97, *P*=.006) compared with age and sex reference data. Patients showed a trend toward improvement in isometric grip force from pre- to postintervention (right: 7% and left: 18%), but it did not reach significance (right: *P*=.16 and left: *P*=.21).

#### Cardiopulmonary Function

Regarding cardiopulmonary function (Table 3), the results of the 6MWT showed a significant increase of 10% in the distance walked from pre- to postintervention. To test whether the increase was clinically significant, a one-sample Wilcoxon signed-rank test analysis showed that the median improvement of 40 m (range 7-159) was significantly different from the threshold of 15 m suggested as the minimal clinically meaningful difference (*P*=.003) [40]. The comparison between the postintervention average distance walked and the reference values was not significant. The preintervention data were not compared with reference values but would most likely not differ because the preintervention walked distance was within the normal range (593 m walked, SD 107 m vs 598 m, SD 43 m).

#### Bone Health

The pre- and postintervention pQCT bone parameter data are shown in Table 4. A significant increase in the following parameters was reported: cortical CSA increased by 4% (14% site; *P*=.001) and 3% (38% site; *P*=.003) and total CSA increased by 2% and 4% at the 14% site (*P*=.01) and 38% site (*P*=.001), respectively. A 6% and 4% increase in SSI was also observed at the 14% site and 38% site (*P*=.001 and *P*=.04), respectively. BMC increased significantly by 4% at the 14% site (*P*=.02) and 38% site (*P*=.003). No other pQCT bone parameters showed significant differences between pre- and postintervention evaluations. To ascertain that changes in bone parameters were associated with exercise training and not entirely to growth, we performed supplementary bivariate correlations between changes in height and weight and changes in bone parameters. No significant association was found between any growth-associated factors and bone parameters (height vs bone CSA at 14%, *P*=.36; height vs bone CSA at 38%, *P*=.28; height vs cortical CSA at 38%, *P*=.74; body weight vs bone CSA at 14%, *P*=.75; body weight vs bone CSA at 38%, *P*=.11; body weight vs cortical CSA at 38%, *P*=.19), suggesting that changes in bone parameters were associated with the mechanical workload of the exercise interventions rather than with growth itself. The comparison of postintervention bone parameters between patients and paired controls revealed that the only bone parameters that were significantly different from controls at the postintervention evaluation were total CSA at the 4% site, which was 21.2% (*P*=.01) larger in patients than in controls, and the SSI at the 14% site, which was 7% (*P*=.007) greater in patients than in controls. No other bone-related significant differences were observed between patients and controls.

The pQCT analysis showed that muscle density, evaluated at the postintervention assessment, was 5% lower (*P*=.05) in patients than in typically developing controls. However, muscle density did not change after the intervention.

**Figure 4:**
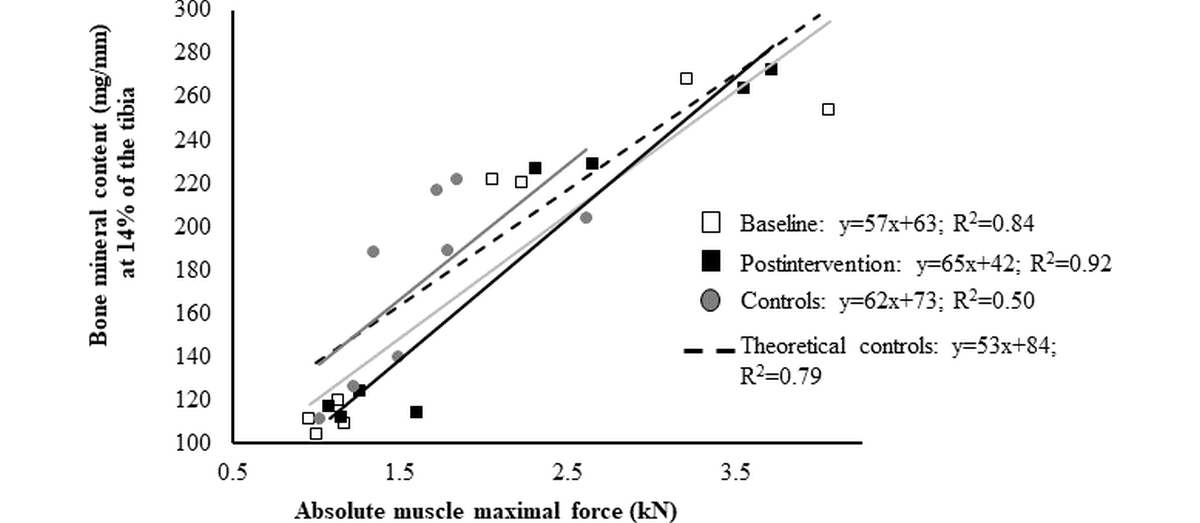
Muscle force and bone strength relationship. Linear correlation between bone mineral content (mg/mm) at the 14% site and muscle force (kN) as a function of the disease status (early-on survivors of acute lymphoblastic leukemia vs age- and sex-matched controls) and testing phase (baseline vs postintervention). Light gray line and white squares depict baseline acute lymphoblastic leukemia data; black line and squares depict postintervention acute lymphoblastic leukemia data. Dark gray line and circles depict age- and sex-matched controls data. Blue line depicts theoretical control muscle-bone relationship.

For the muscle-bone functional unit (Figure 4), there was a significant positive relationship between absolute peak force and BMC at 14% at preintervention (*P*=.01), at postintervention (*P*=.004), and for controls (*P*=.007). The slopes were similar for both patient slopes within 10% of the controls.

**Table 4:** Bone health parameters assessed with peripheral quantitative computed tomography.

Muscle and bone health parameters			Baseline evaluation		Postintervention evaluation		Controls		*P* value^a^		*P* value^b^
**Calf muscle (n=8)^c^**											
	Muscle CSA^d^ (mm^2^)^e^, median (range)	3503 (2687-7222)		3606 (2766-6921)		4618 (2966-6828)		.26		.86	
	Muscle density (mg/cm^3^), mean (SD)	68.7 (4.0)		68.6 (3.3)		71.9 (1.8)		.97		*.05* ^f^	
**Tibia 4% site (n=9)**											
	Total CSA (mm^2^), mean (SD)	819 (325)		830 (332)		675 (227)		.12		*.01*	
	Total BMC^g^ (mg/mm), mean (SD)	234 (99)		239 (103)		207 (58)		.17		.10	
	Total vBMD^h^ (mg/cm^3^), mean (SD)	289.46 (37)		289 (32)		312 (31)		.98		.14	
	Trabecular vBMD (mg/cm^3^), mean (SD)	218.92 (38)		216 (38)		212 (19)		.53		.77	
**Tibia 14% site (n=8)^c^**											
	Total CSA^e^ (mm^2^), median (range)	341 (188-517)		349 (194-528)		294 (188-406)		*.01*		.38	
	Total BMC^e^ (mg/mm), median (range)	170 (104-268)		175 (112-272)		188 (111-222)		*.03*		.72	
	Cortical CSA (mm^2^), mean (SD)	120 (44)		124 (44)		133 (34)		*.001*		.68	
	Cortical vBMD (mg/cm^3^), mean (SD)	1000 (32)		1004 (30)		994 (50)		.26		.62	
	SSI^i^ (mm^3^), mean (SD)	891 (510)		932 (519)		837 (310)		*.001*		*.007*	
**Tibia 38% site (n=9)**											
	Total CSA (mm^2^), mean (SD)	292 (116)		301 (116)		282 (85)		*.001*		.47	
	Total BMC (mg/mm), mean (SD)	222 (78)		229 (79)		233 (62)		*.003*		.89	
	Cortical CSA (mm^2^), mean (SD)	191 (70)		198 (71)		206 (53)		*.003*		.86	
	Cortical vBMD^e^ (mg/cm^3^), median (range)	1056 (927-1088)		1045 (930-1096)		1036 (959-1094)		.86		.80	
	SSI (mm^3^), mean (SD)	906 (498)		939 (507)		948 (387)		*.04*		.37	

^a^*P* values of paired-sample *t* test and Wilcoxon matched-pairs signed-ranked test comparing baseline and postintervention parameters.

^b^*P* values of independent-samples *t* test and independent-samples Mann-Whitney *U* test comparing postintervention data with age- and sex-matched controls’ data.

^c^One peripheral quantitative computed tomography scan removed due to movement artifact.

^d^CSA: cross-sectional area.

^e^Parameters not normally distributed.

^f^Italicized values indicates significance of *P*<.05.

^g^BMC: bone mineral content.

^h^vBMD: volumetric bone mineral density.

^i^SSI: stress-strain index.

## Discussion

### Principal Findings

The primary objective of this study was to assess the feasibility of administering a supervised telehealth home-based exercise intervention for early-on survivors of acute lymphoblastic leukemia. The hypothesis was confirmed, and the approach was deemed feasible, as demonstrated by the 75% completion rate and mean adherence rate of 89%. The secondary objective of this study was to explore the benefits of exercise interventions on functional outcomes and bone health parameters. In line with our exploratory hypothesis, an improvement in lower limb muscle function and bone health parameters was observed between pre- and postintervention evaluations.

### Feasibility

Compared with an unsupervised home-based exercise intervention, the adherence and completion rates (89% and 75%, respectively) reported in this study are high [15], suggesting that direct supervision contributes to a high adherence rate. These numbers are similar to those reported by Esbenshade et al [20], who reported adherence rates of 81% and completion rates of 71%. This study and a study by Esbenshade et al [20] showed similar improvements in muscle and cardiopulmonary function. In the study by Esbenshade et al [20], direct supervision was not provided, but weekly follow-up phone calls were made. Direct supervision has many advantages that can lead to increased adherence and participation rates [23]. Receiving positive reinforcement [24], improving exercise technique [24], and feeling self-efficient [25] are all benefits associated with increased adherence and, indirectly, health benefits. Another positive impact of supervision is the greater overall volume of exercise achieved during individual sessions, which can be associated with better structured and controlled exercised sessions under supervision compared with no supervision or phone call follow-up [26]. In this study, having direct supervision might have led to more efficient training because many sessions needed exercise adaptations because of patient pain. Being able to adjust the exercises to avoid pain in real time may have prevented injuries that may have prevented a decrease in participation and adherence rates. According to our data, the high proportion of training that required adaptation for pain management would suggest the need for direct supervision for safety purposes for this specific patient population prone to musculoskeletal-related pain [49,50].

A favorable aspect of the telehealth approach used in this study is that patients showed similar improvement in musculoskeletal and cardiopulmonary functions as in other studies using indirect supervision (phone calls and video recordings of the exercises to be performed) but with lower volume and frequency. In a study by Tanir and Kuguoglu [19], muscle function training was required 3 days per week, 3 times per day, in addition to 3 times per week, once a day of aerobic training, whereas in a study by Esbenshade et al [20], resistance training was required three times per week and aerobic training was required three times per week, for a total training time ranging between 3.5 and 5.25 hours per week. In comparison, the weekly amount of time devoted to training in our study reached a maximum of 2.25 hours (3×45 min). Taken together, this suggests that having a qualified kinesiologist supervising the training sessions improves the efficiency with which the patients are performing the training [26].

### Functional Performance and Bone Health

Although this study aimed to evaluate feasibility, it was hypothesized that improvements would be observed for muscle, bone, and cardiopulmonary fitness parameters. Regarding muscle parameters, a previous study in a patient population showing similar muscle weaknesses established [33] the minimal detectable difference to be of 0.42 multiples of body weight for relative force (M2LH test) and 3.19 W/kg for relative power (S2LJ). In this study, and once the patient who relapsed was removed from the analysis, the improvements were 0.55 multiples of body weight for relative force and 3.05 W/kg for relative power. Notably, all patients showed improvements in these parameters. These results are similar to the reported minimal detectable difference, suggesting clinically relevant improvements in our patients.

In terms of cardiopulmonary fitness, the 6MWT walking distance showed an increase of 53 m from pre- to postintervention. This is more than the 15-m the minimal clinically meaningful difference reported in a previous study evaluating the between-session reproducibility of the 6MWT walking distance [40]. This suggests that the improvement represents true changes rather than the measurement variability.

At the bone level, the mechanostat theory, developed by Frost [51], stipulates that bones adapt to maximal mechanical loading applied from muscle contractions and unfavorable lever arms. In this study, a special emphasis was placed on increasing lower limb muscle force and, indirectly, mechanical loading of patients’ bones. The results indicated significant improvements in multiple bone-related parameters, such as BMC and cross-section. Figure 4 shows that the linear relationship associated with muscle force and bone strength parameters was normal in the patient population with acute lymphoblastic leukemia and maintained postintervention. These results suggest that the bone mechanotransduction and modeling process are normal in young early-on survivors of hematological malignancies and that the intervention aimed at increasing muscle force may lead to increased bone strength [44].

### Limitations of the Study

In total, two major challenges in the recruitment process were identified: (1) only one-fifth of the patients who were contacted provided informed consent (12/57, 21%) and (2) creating the groups revealed to be difficult, leading to multiple cohorts. Regarding the families that declined participation, the four families that declined because patients were too active were more advanced in their survivorship (>2 years) and returned to their daily living activities before the diagnosis. This suggests that implementing a home-based exercise intervention may be more feasible earlier (1-2 years) than later (3-5 years) in their survivorship. The group approach was also challenging because of two factors: (1) the age range of the participants and (2) the availabilities of families leading to bilingual groups. Owing to difficulty in recruiting patients, the protocol was amended to increase the age range of the study participants from 6 to 10 years to 6 to 18 years. This resulted in one group having two 9-year-old training with a 14-year-old patient, which is not ideal. Québec has a very large proportion of French- and English-speaking population, and both are represented in this study. English- and French-speaking participants had to be grouped together because of family availability constraints, leading to providing bilingual training sessions.

Owing to administrative constraints, the intervention was offered to participants approximately 2 weeks before the start of the interventions. To avoid the challenges mentioned earlier, it is recommended that such interventions for early-on survivors be offered to families during routine checkups, months before their participation. This would provide families sufficient time to organize their schedules to integrate the training intervention with school activities and other obligations. This would also provide the clinician with the opportunity to avoid recruitment pitfalls and the challenges reported earlier.

The positive impact of mobile technology on families’ experiences was unforeseen; hence, limited results have been reported. This project was originally designed to be a traditional home-based intervention; however, mobile technology allowed accessibility to the interventions outside the household, favoring everyday life activities versus exercise training balance. As such, families took full opportunity to use their own technologies to train in different environments, such as on their family vacations at the hotel or during family dinners. Without mobile technologies, families would have had to choose between attending training session or their social events. Similarly, the clinician was able to deliver interventions while on a scientific conference travel outside the country over a 2-week period. Nonetheless, the use of personal technologies can be disadvantageous to some families with lower socioeconomic status, which may have limited access to personal technologies and high-speed internet. In this study, no patients declined to participate because of a lack of accessible information technology, but one family was excluded because the available internet connection in their geographical area was too unstable to allow communication and safe exercise supervision.

In terms of health benefits, the results of the intervention are promising, showing improvements in most muscle, cardiopulmonary fitness, and bone measured parameters. However, this study was designed to assess the feasibility of a home-based exercise intervention delivered through telehealth; therefore, it was neither powered nor designed to detect the physiological changes associated with the intervention. In this regard, functional performance and bone health results were analyzed simply and may have statistical artifacts, such as the multiple hypothesis testing effect, because no statistical corrections were applied. Improvements in functional performance and bone health should thus be interpreted with these considerations in mind, despite the fact that improvement was observed in 8 of 9 study participants. Another limitation of this study is that the control group consisted of healthy individuals, rather than early-on survivors who would not receive the intervention. This prevents any conclusion regarding whether the proposed approach provides added benefits compared with the standard of care. The second limitation is associated with the retrospective nature of the control group, which only had data for one time point. This prevented performing adequate statistical analyses comparing the two groups pre- and postintervention. Therefore, any control group comparison should be interpreted in this context.

### Conclusions

The results of this study suggest that providing early-on survivors of acute lymphoblastic leukemia with home-based exercise intervention through telehealth is a feasible approach. This approach has multiple advantages, even more so in the context of the current COVID-19 pandemic. Patients with acute lymphoblastic leukemia are usually treated in specialized (tertiary) health care centers located in large cities. As a result, patients treated at these centers are scattered across large distances, making the implementation of frequent adjunct therapies impossible. Finally, although exploratory in nature, the comparison between pre- and postintervention muscle and bone parameters suggests that the proposed exercise regimen is suitable for inducing musculoskeletal benefits in young early-on survivors of bone marrow–related hematological malignancies.

## Abbreviations

6MWT: 6-minute walk test
BMC: bone mineral content
CSA: cross-sectional area
M2LH: multiple two-legged hopping
pQCT: peripheral quantitative computed tomography
SSI: stress-strain index
S2LJ: single two-legged jump
vBMD: volumetric bone mineral density

## References

[1] Siegel DA, Claridy M, Mertens A, George E, Vangile K, Simoneaux SF, Meacham LR, Wasilewski-Masker K (2017). Risk factors and surveillance for reduced bone mineral density in pediatric cancer survivors. Pediatr Blood Cancer.

[2] Lemay V, Caru M, Samoilenko M, Drouin S, Mathieu M, Bertout L, Lefebvre G, Raboisson M, Krajinovic M, Laverdière C, Andelfinger G, Sinnett D, Curnier D (2020). Physical activity and sedentary behaviors in childhood acute lymphoblastic leukemia survivors. J Pediatr Hematol Oncol.

[3] Warner JT (2008). Body composition, exercise and energy expenditure in survivors of acute lymphoblastic leukaemia. Pediatr Blood Cancer.

[4] Mostoufi-Moab S, Ward LM (2019). Skeletal morbidity in children and adolescents during and following cancer therapy. Horm Res Paediatr.

[5] Marchese VG, Chiarello LA, Lange BJ (2003). Strength and functional mobility in children with acute lymphoblastic leukemia. Med Pediatr Oncol.

[6] Atkinson SA, Halton JM, Bradley C, Wu B, Barr RD (1998). Bone and mineral abnormalities in childhood acute lymphoblastic leukemia: influence of disease, drugs and nutrition. Int J Cancer Suppl.

[7] Boot AM, van den Heuvel-Eibrink MM, Hählen K, Krenning EP, de Muinck KS (1999). Bone mineral density in children with acute lymphoblastic leukaemia. Eur J Cancer.

[8] Alos N, Grant RM, Ramsay T, Halton J, Cummings EA, Miettunen PM, Abish S, Atkinson S, Barr R, Cabral DA, Cairney E, Couch R, Dix DB, Fernandez CV, Hay J, Israels S, Laverdière C, Lentle B, Lewis V, Matzinger M, Rodd C, Shenouda N, Stein R, Stephure D, Taback S, Wilson B, Williams K, Rauch F, Siminoski K, Ward LM (2012). High incidence of vertebral fractures in children with acute lymphoblastic leukemia 12 months after the initiation of therapy. J Clin Oncol.

[9] Halton J, Gaboury I, Grant R, Alos N, Cummings EA, Matzinger M, Shenouda N, Lentle B, Abish S, Atkinson S, Cairney E, Dix D, Israels S, Stephure D, Wilson B, Hay J, Moher D, Rauch F, Siminoski K, Ward LM, Canadian STOPP Consortium (2009). Advanced vertebral fracture among newly diagnosed children with acute lymphoblastic leukemia: results of the Canadian Steroid-Associated Osteoporosis in the Pediatric Population (STOPP) research program. J Bone Miner Res.

[10] Mueske NM, Mittelman SD, Wren TAL, Gilsanz V, Orgel E (2019). Myosteatosis in adolescents and young adults treated for acute lymphoblastic leukemia. Leuk Lymphoma.

[11] Orgel E, Mueske NM, Wren TA, Gilsanz V, Butturini AM, Freyer DR, Mittelman SD (2016). Early injury to cortical and cancellous bone from induction chemotherapy for adolescents and young adults treated for acute lymphoblastic leukemia. Bone.

[12] Marriott CJ, Beaumont LF, Farncombe TH, Cranston AN, Athale UH, Yakemchuk VN, Webber CE, Barr RD (2018). Body composition in long-term survivors of acute lymphoblastic leukemia diagnosed in childhood and adolescence: a focus on sarcopenic obesity. Cancer.

[13] Watsky MA, Carbone LD, An Q, Cheng C, Lovorn EA, Hudson MM, Pui C, Kaste SC (2014). Bone turnover in long-term survivors of childhood acute lymphoblastic leukemia. Pediatr Blood Cancer.

[14] Behringer M, Gruetzner S, McCourt M, Mester J (2014). Effects of weight-bearing activities on bone mineral content and density in children and adolescents: a meta-analysis. J Bone Miner Res.

[15] Manchola-González JD, Bagur-Calafat C, Girabent-Farrés M, Serra-Grima JR, Pérez RA, Garnacho-Castaño MV, Badell I, Ramírez-Vélez R (2020). Effects of a home-exercise programme in childhood survivors of acute lymphoblastic leukaemia on physical fitness and physical functioning: results of a randomised clinical trial. Support Care Cancer.

[16] Via JD, Daly RM, Fraser SF (2018). The effect of exercise on bone mineral density in adult cancer survivors: a systematic review and meta-analysis. Osteoporos Int.

[17] Wright M (2015). Physical activity participation and preferences: developmental and oncology-related transitions in adolescents treated for cancer. Physiother Can.

[18] Ross WL, Le A, Zheng DJ, Mitchell H, Rotatori J, Li F, Fahey JT, Ness KK, Kadan-Lottick NS (2018). Physical activity barriers, preferences, and beliefs in childhood cancer patients. Support Care Cancer.

[19] Tanir MK, Kuguoglu S (2013). Impact of exercise on lower activity levels in children with acute lymphoblastic leukemia: a randomized controlled trial from Turkey. Rehabil Nurs.

[20] Esbenshade AJ, Friedman DL, Smith WA, Jeha S, Pui C, Robison LL, Ness KK (2014). Feasibility and initial effectiveness of home exercise during maintenance therapy for childhood acute lymphoblastic leukemia. Pediatr Phys Ther.

[21] Marchese VG, Chiarello LA, Lange BJ (2004). Effects of physical therapy intervention for children with acute lymphoblastic leukemia. Pediatr Blood Cancer.

[22] Hartman A, te Winkel M, van Beek R, de Muinck KS, Kemper H, Hop W, van den Heuvel-Eibrink M, Pieters R (2009). A randomized trial investigating an exercise program to prevent reduction of bone mineral density and impairment of motor performance during treatment for childhood acute lymphoblastic leukemia. Pediatr Blood Cancer.

[23] Argent R, Daly A, Caulfield B (2018). Patient involvement with home-based exercise programs: can connected health interventions influence adherence?. JMIR Mhealth Uhealth.

[24] Marshall A, Donovan-Hall M, Ryall S (2012). An exploration of athletes' views on their adherence to physiotherapy rehabilitation after sport injury. J Sport Rehabil.

[25] Bassett S (2015). Bridging the intention-behaviour gap with behaviour change strategies for physiotherapy rehabilitation non-adherence. NZ J Physiother.

[26] Stout NL, Baima J, Swisher AK, Winters-Stone KM, Welsh J (2017). A systematic review of exercise systematic reviews in the cancer literature (2005-2017). PM R.

[27] Horsley S, Schock G, Grona SL, Montieth K, Mowat B, Stasiuk K, Boden C, Bath B (2020). Use of real-time videoconferencing to deliver physical therapy services: a scoping review of published and emerging evidence. J Telemed Telecare.

[28] van Egmond MA, van der Schaaf M, Vredeveld T, Vollenbroek-Hutten MM, van Berge Henegouwen MI, Klinkenbijl JH, Engelbert RH (2018). Effectiveness of physiotherapy with telerehabilitation in surgical patients: a systematic review and meta-analysis. Physiotherapy.

[29] Lambert G, Drummond K, Ferreira V, Carli F (2021). Teleprehabilitation during COVID-19 pandemic: the essentials of "what" and "how". Support Care Cancer.

[30] Kraemer WJ, Ratamess NA (2004). Fundamentals of resistance training: progression and exercise prescription. Med Sci Sports Exerc.

[31] Birnie KA, Hundert AS, Lalloo C, Nguyen C, Stinson JN (2019). Recommendations for selection of self-report pain intensity measures in children and adolescents: a systematic review and quality assessment of measurement properties. Pain.

[32] Ory M, Resnick B, Jordan PJ, Coday M, Riebe D, Ewing GC, Pruitt L, Bazzarre T (2005). Screening, safety, and adverse events in physical activity interventions: collaborative experiences from the behavior change consortium. Ann Behav Med.

[33] Veilleux L, Lemay M, Pouliot-Laforte A, Cheung MS, Glorieux FH, Rauch F (2014). Muscle anatomy and dynamic muscle function in osteogenesis imperfecta type I. J Clin Endocrinol Metab.

[34] Veilleux L, Rauch F (2010). Reproducibility of jumping mechanography in healthy children and adults. J Musculoskelet Neuronal Interact.

[35] Anliker E, Rawer R, Boutellier U, Toigo M (2011). Maximum ground reaction force in relation to tibial bone mass in children and adults. Med Sci Sports Exerc.

[36] Robinson M, Bardai G, Veilleux L, Glorieux FH, Rauch F (2020). Musculoskeletal phenotype in two unrelated individuals with a recurrent nonsense variant in SGMS2. Bone.

[37] Wong SL (2016). Grip strength reference values for Canadians aged 6 to 79: Canadian Health Measures Survey, 2007 to 2013. Health Rep.

[38] Hooke MC, Garwick AW, Neglia JP (2013). Assessment of physical performance using the 6-minute walk test in children receiving treatment for cancer. Cancer Nurs.

[39] Mizrahi D, Fardell JE, Cohn RJ, Partin RE, Howell CR, Hudson MM, Robison LL, Ness KK, McBride J, Field P, Wakefield CE, Simar D (2020). The 6-minute walk test is a good predictor of cardiorespiratory fitness in childhood cancer survivors when access to comprehensive testing is limited. Int J Cancer.

[40] Li AM, Yin J, Yu CC, Tsang T, So HK, Wong E, Chan D, Hon EK, Sung R (2005). The six-minute walk test in healthy children: reliability and validity. Eur Respir J.

[41] Labonté J, Caru M, Lemay V, Alos N, Drouin S, Bertout L, Andelfinger G, Krajinovic M, Laverdière C, Sinnett D, Curnier D (2020). Developing and validating equations to predict [Formula: see text]O2 peak from the 6MWT in Childhood ALL Survivors. Disabil Rehabil.

[42] Ulrich S, Hildenbrand FF, Treder U, Fischler M, Keusch S, Speich R, Fasnacht M (2013). Reference values for the 6-minute walk test in healthy children and adolescents in Switzerland. BMC Pulm Med.

[43] Morinder G, Mattsson E, Sollander C, Marcus C, Larsson UE (2009). Six-minute walk test in obese children and adolescents: reproducibility and validity. Physiother Res Int.

[44] Veilleux L, Pouliot-Laforte A, Lemay M, Cheung MS, Glorieux FH, Rauch F (2015). The functional muscle-bone unit in patients with osteogenesis imperfecta type I. Bone.

[45] Veilleux L, Cheung MS, Glorieux FH, Rauch F (2013). The muscle-bone relationship in X-linked hypophosphatemic rickets. J Clin Endocrinol Metab.

[46] Rittweger J, Beller G, Ehrig J, Jung C, Koch U, Ramolla J, Schmidt F, Newitt D, Majumdar S, Schiessl H, Felsenberg D (2000). Bone-muscle strength indices for the human lower leg. Bone.

[47] Blew RM, Lee VR, Farr JN, Schiferl DJ, Going SB (2014). Standardizing evaluation of pQCT image quality in the presence of subject movement: qualitative versus quantitative assessment. Calcif Tissue Int.

[48] Ghasemi A, Zahediasl S (2012). Normality tests for statistical analysis: a guide for non-statisticians. Int J Endocrinol Metab.

[49] Arpaci T, Toruner EK (2016). Assessment of problems and symptoms in survivors of childhood acute lymphoblastic leukaemia. Eur J Cancer Care (Engl).

[50] Haddy TB, Mosher RB, Reaman GH (2001). Osteoporosis in survivors of acute lymphoblastic leukemia. Oncologist.

[51] Frost HM (2003). Bone's mechanostat: a 2003 update. Anat Rec A Discov Mol Cell Evol Biol.

